# Malnutrition Prevalence and Nutrient Intakes of Indonesian Community-Dwelling Older Adults: A Systematic Review of Observational Studies

**DOI:** 10.3389/fnut.2022.780003

**Published:** 2022-02-24

**Authors:** Esthika Dewiasty, Rina Agustina, Siti Rizny F. Saldi, Arvin Pramudita, Fenna Hinssen, Meutia Kumaheri, Lisette C. P. G. M. de Groot, Siti Setiati

**Affiliations:** ^1^Department of Internal Medicine, Cipto Mangunkusumo Hospital, Faculty of Medicine, Universitas Indonesia, Jakarta, Indonesia; ^2^Division of Human Nutrition and Health, Wageningen University and Research, Wageningen, Netherlands; ^3^Department of Nutrition, Cipto Mangunkusumo Hospital, Faculty of Medicine, Universitas Indonesia, Jakarta, Indonesia; ^4^Human Nutrition Research Center, Indonesian Medical Education and Research Center, Faculty of Medicine, Universitas Indonesia, Jakarta, Indonesia; ^5^Center of Clinical Epidemiology and Evidence-Based Medicine, Cipto Mangunkusumo Hospital, Faculty of Medicine, Universitas Indonesia, Jakarta, Indonesia

**Keywords:** malnutrition, macronutrients, micronutrients, inadequacies, community-dwelling, older adults, Indonesia

## Abstract

**Background:**

Malnutrition and inadequate nutrient intake are associated with functional decline, frailty, and bad clinical outcomes among community-dwelling older adults. Despite the growing proportion of the elderly population in Indonesia, data on the prevalence of malnutrition and adequacy of macronutrient and micronutrient intakes among Indonesian older adults are scattered and vary between studies. Therefore, our study aims to obtain data on malnutrition prevalence, level and distribution of nutrient intakes, and prevalence of macronutrient and micronutrient inadequacies in Indonesian community-dwelling older adults.

**Methods:**

We carried out a systematic review following the Preferred Reporting Items for Systematic Reviews and Meta-Analyses statement and registered in PROSPERO. A systematic electronic database search of MEDLINE, CENTRAL, EMBASE, ProQuest, HINARI, IMSEAR, GARUDA, and Indonesian Publication Index was undertaken. Additional searches were conducted in gray literature sources, hand-searching, retrospective searching, and personal communication with authors of the relevant publication. Observational studies presenting the malnutrition prevalence of habitual dietary intakes of older adults (60 years or older) were included. The risk of bias of studies was assessed using the Joanna Briggs Institute critical appraisal form. Sex-specific mean (and standard deviation) habitual macronutrient and a selection of micronutrients (calcium, vitamin D, and vitamin B12) intakes were extracted from each article to calculate the percentage of older people who were at risk for inadequate micronutrient intakes using a proxy of estimated average requirement (EAR) cut-point method, which is calculated from the national guideline of recommended dietary allowance (RDA). Prevalence of malnutrition, based on body mass index (BMI) categories and mini-nutritional assessment (MNA) criteria. and the population at risk of malnutrition were presented descriptively.

**Results:**

Nine studies retrieved from electronic databases and gray literature were included in the pooled systematic analysis. According to BMI criteria, the underweight prevalence ranged from 8.0 to 26.6%. According to the MNA, the prevalence of malnutrition ranged from 2.1 to 14.6%, whereby the prevalence of at risk of malnutrition amounted to 18–78%. Our systematic review identified a high prevalence of nutrient inadequacies, most markedly for protein, calcium, vitamin D, and vitamin B12.

**Conclusion:**

We signal a high risk of malnutrition along with poor macronutrients and micronutrients intakes among Indonesian community-dwelling older adults. These findings provide important and robust evidence on the magnitude of malnutrition and nutrient inadequacy concerns that call for appropriate nutrition, as well as public health policies and prompt intervention.

**Systematic Review Registration:**

https://www.crd.york.ac.uk/prospero/display_record.php?ID=CRD42018102268.

## Introduction

The world's population is aging. In recent years, the elderly population is growing rapidly in developed and developing countries, including Indonesia. In Indonesia, more than 7% of 250 million residents is over the age of 60. This percentage is higher than it was in 1980 when it amounted to 5.4% (1). In Indonesia, the life expectancy is expected to increase from 67.8 years in 2000–2005 to 73.6 years in 2020–2025. Ranking 6th as the most aging population globally, Indonesia faces an increasing prevalence of several aging-related diseases, including malnutrition (2). As underlined by ESPEN, nutrition is an important modulator of health and wellbeing in older adults (3). Inadequate nutrition contributes to the progression of many diseases and is regarded as an important contributing factor in the complex etiology of sarcopenia and frailty. A close relation between malnutrition and poor outcomes, e.g., increased rates of infections and pressure ulcers, increased length of hospital stay, increased duration of convalescence after acute illness, and increased mortality, is well-documented in older adults (3). The Malnutrition in the Elderly (MaNuEL) Knowledge Hub signaled that malnutrition is also associated with a negative impact on the wellbeing and quality of life of the older individual, as well as with increased healthcare costs (4). Thus, malnutrition (especially undernutrition) has been considered both a geriatric syndrome (5) and an important public health concern (6). Hereby, low energy and nutrient intake reflect one of the major mechanisms defining the potential determinants of malnutrition: inadequate intake, high requirements, and impaired bioavailability of energy and nutrients.

Despite the growing proportion of the elderly population in Indonesia, data on the prevalence of malnutrition and low nutrient intake among Indonesian older adults are scattered and vary between studies (7). A large population-based survey would be the most valuable way to answer a prevalence question, addressing both malnutrition and the inadequacies of dietary intakes, thereby also collecting data on the participants' characteristics and their health/disease status. However, efforts to initiate such a survey haven't been successful and apparently it is not given priority in pandemic times. Thereby, a large national nutrition and health survey is costly, time-consuming, and frequently impractical. A systematic review of prevalence studies (of both malnutrition and dietary inadequacies), on the other hand, has a number of advantages that are relevant to our situation in terms of feasibility, comprehensiveness of the available evidence, providing information for further research priorities, and providing data for policy makers (8, 9). To our knowledge, apart from unpublished data found in the gray literature, there are very few published studies reporting on the prevalence of malnutrition and adequacy of nutrient intake in Indonesian older adults. Thus, for a comprehensive review, the gray literature might be an important source. Hence, we conducted a systematic review including both open and gray literature, aiming to estimate (1) the prevalence of malnutrition, (2) the level and distribution of habitual intakes of energy and nutrients, and (3) the prevalence of inadequate intakes of energy and nutrients among Indonesian community dwelling older adults.

## Materials and Methods

This systematic review was registered on the 24th of July 2018 in the international prospective register of systematic review (PROSPERO): CRD42018102268, and was written according to the Preferred Reporting Items for Systematic Reviews and Meta-Analysis (PRISMA) checklist (10).

### Search Strategy

We pre-defined the data items before searching, as described in Appendix 1. A systematic electronic database search of MEDLINE, CENTRAL, EMBASE, ProQuest, HINARI, IMSEAR, GARUDA, and Indonesian Publication Index was undertaken from inception to May 2019 by two investigators (SRF and AP), and updated in September 2020 to retrieve all observational studies in Indonesian, geriatric, community-dwelling population. These terms were combined with “OR” and “AND” operators. The Medical Subject Headings (MeSH) terms include geriatrics, frail older adults, malnutrition, cachexia, undernutrition, underweight, energy intake, dietary carbohydrate, dietary protein, dietary fats, micronutrients. Energy intake refers to daily dietary energy. Macronutrients included in this study comprise carbohydrates, protein, fat, saturated fatty acid (SFA), mono-unsaturated fatty acid (MUFA), and poly-unsaturated fatty acid (PUFA). While micronutrients included were vitamin D, vitamin B12, and calcium. The search was targeting results of observational studies: preferably primary cross-sectional, cohort, or case-control studies. However, editorials, letters, opinion articles, narrative or systematic reviews, brief communications, conference abstracts, and posters were also screened. Additional searches were conducted in gray literature sources such as universities' repositories, government websites, and data from the Indonesian Geriatrics Society (PERGEMI). Hand-searching, retrospective searching, and personal communication with authors of relevant published literature were also undertaken. The full search strategy is provided in the **Supplementary Material** (Appendix 1).

### Study Selection

After the search was performed, studies were independently screened by two authors (ED and RSF) on titles and abstracts to assess eligibility based on population and outcomes. The screening took place following predefined selection criteria: Studies were eligible if they contained data on the prevalence of malnutrition or nutrient intake data, participants' age was ≥60 years, and had data originating from Indonesian populations. Due to the scarcity of data and insubstantial difference between older adults and pre-older adults, we made an amendment to PROSPERO regarding the definition of our population: older adults were defined as people aged 60 years or older. We also considered studies which enrolled participants 50–59 year old age as pre-older adults. The title and abstract of the studies were screened to remove duplicates, animal studies, *in vitro* studies, intervention studies, review articles, in-hospital settings, and populations other than Indonesian. Any disagreements were resolved through discussion between the two researchers (ED and SRF). The study selection was performed using Covidence^®^.

### Eligibility Criteria

The Indonesian community-dwelling older adult population was the target population of this review. As age cut-offs for “older adults” varied across studies, a cut-off of 60 years or older for older adults, and 50–59 years old for pre-older adults was chosen to enable a broad inclusion. Community-dwelling older adults were defined as those living at home, living in private households, independently living, free-living, or non-institutionalized (11). Malnutrition was assessed in several ways. We put priority on studies that applied the MNA (long or short form). Yet, we also acknowledged the consented definition and diagnosis of malnutrition based on markedly low body mass index (BMI) (6). Multiple publications were generated from the same data with different outcomes, including energy, protein, fat, macronutrients, and micronutrients; each of those publications was included. However, if several publications reported outcomes from the same study population, only the most relevant publication based on sample size and characteristics of subjects was included. There was no restriction in terms of year of publication and language.

### Quality Assessment

Quality assessment comprising critical appraisal and risk of bias of the individual full-text articles was performed independently by three independent reviewers (LDG, SS, and ED) making use of the JBI critical appraisal checklist for studies reporting prevalence data (Appendix 2) (12). The critical appraisal form assesses the sample representativeness of the target population, recruitment method of study participants, adequacy of sample size, description of study subjects and setting, method of data analysis, standard criteria used for measurement, reliable measurement of outcome, appropriate statistical analysis, identification and handling of important confounding factors, as well as identification of subpopulations using objective criteria. Any disagreements were resolved by discussions between at least two out of three authors: ED, LdG, or SS.

### Data Extraction

The JBI data extraction form for prevalence studies (Appendix 3) was used to extract all relevant data from the articles that were included by two authors separately (ED and FH). Extracted information included study design, study setting, participant characteristics, measurements performed, and results. The published outcomes concern the prevalence of malnutrition in percentage and the habitual intake of energy and nutrients in provided units. The data extraction results by the two authors have been combined afterward, and any disagreement was resolved by discussion between ED and FH. If necessary, the two authors will seek third opinion from LdG, SS, or RA.

### Summary Measures

Data on the prevalence of malnutrition as well as the level, distribution, and prevalence of inadequacies of habitual intake of energy, macronutrients, and micronutrients were described in summary tables. Prevalence was presented as a percentage. For each of the included studies, the mean (and standard deviation) habitual dietary intakes of macronutrients and micronutrients were extracted. When necessary, means and measures of dispersion were estimated from the figures in the reports. Standard errors of nutrient intake as published in some studies were converted standard deviations by using the derivation formula of standard error = standard deviation/√ (sample size) beforehand (13), to make the data more homogenous and available for further analysis (14).

To assess the prevalence of inadequacies, the reported mean intakes were compared to the recommended dietary intakes of the Indonesian population (Indonesian RDA). The Indonesian RDA for age group 65–80 years old was used as the dietary reference intake. The results of nutrient intake of all of the studies included in this review were compared to the RDA, as most Asian countries did and as estimated average requirements (EAR) values are lacking (15). Furthermore, we also calculated the prevalence of inadequacies based on 2/3 RDA, as a proxy of EAR, as we will use it as a value of DRI in this study (15, 16).

We calculated the prevalence of inadequacy using the following calculation: z = (x–μ)/SD, with x as the dietary reference intake, μ the mean nutrient intake, and SD the standard deviation of the nutrient intake (17). Then, we calculated the percentage below dietary reference intake based on its corresponding value in the Z-scores table (18). To determine the prevalence of inadequacies, we compared nutrient intakes data with the national nutritional reference values presented as RDA age 65–80 (Appendix 4) (19), and 2/3 of this RDA, assumed as proxy of the EAR (15, 16).

## Results

### Study Selection

**Figure 1:**
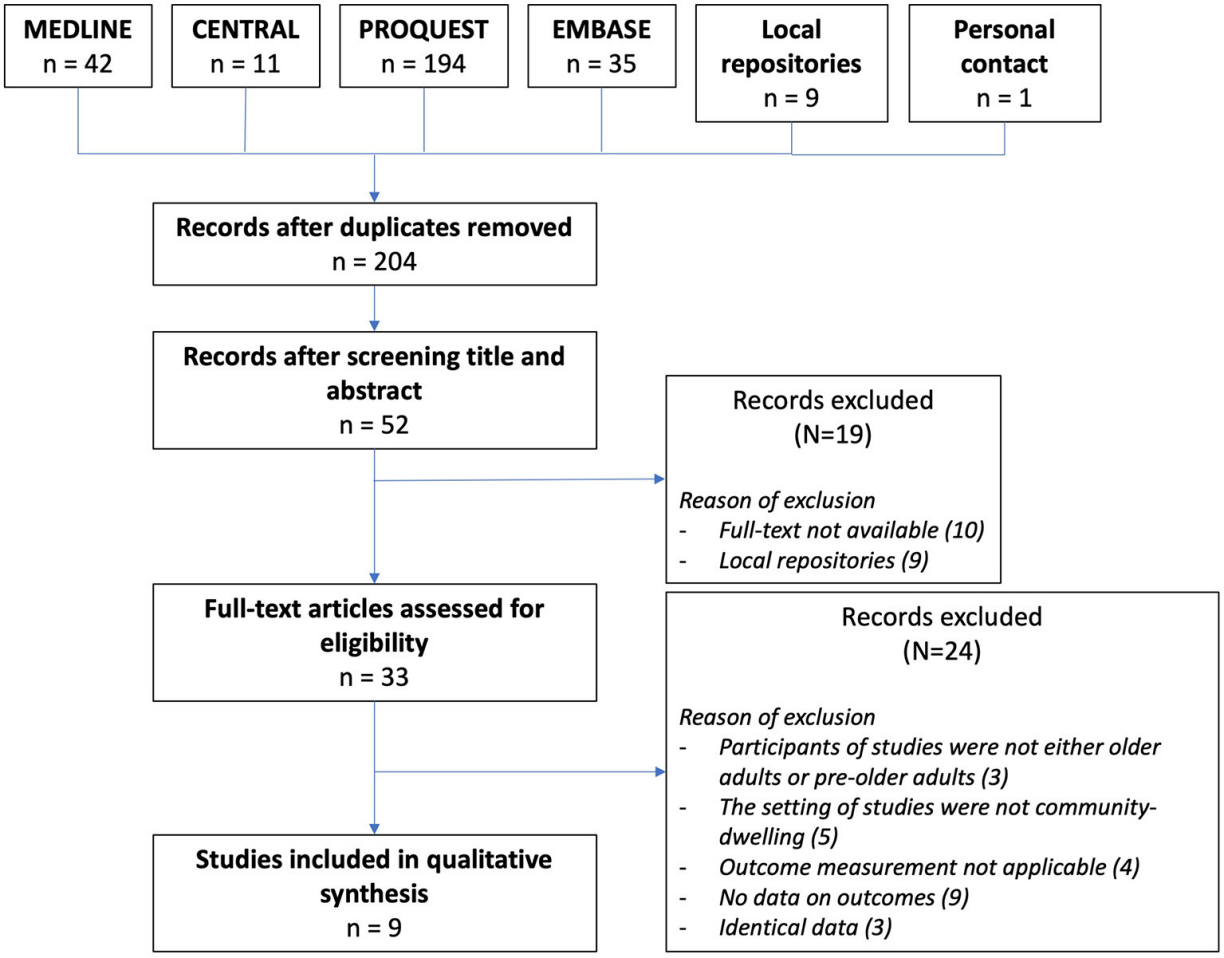
PRISMA flow chart of the searching method resulting in 9 studies included in the systematic review.

The search resulted in a total of 291 publications from several electronic databases. Nine of these publications were eligible, one of them (20) was retrieved after the protocol amendment. One additional study from the gray literature (personal communication with authors in Universitas Indonesia repository) was added. Figure 1 shows the literature search process with the reason of the exclusion of 43 articles.

### Study Characteristics of Included Studies

Characteristics of the nine included studies are presented in Table 1. The sample size ranges from 58 to 3585 elderly subjects. By population, the included studies vary from nationwide studies Purba and Pengpid (22, 24), multicenter outpatients' studies (8, 26, 27), and specific areas, e.g., rural, sub-urban, or urban areas (20, 21, 23, 25). Nutrient intakes were assessed by either FFQ (21, 22, 24, 25), 24-h recall (8, 20), or both methods (23). Two studies (26, 27) did not mention the methods of dietary intake assessment. Methods to assess nutritional status include BMI, anthropometrics, MNA (full form and short form), and chemical biomarkers.

**Table 1:** Descriptive characteristics of the included studies.

**No**	**References**	**Number of subjects**	**Aims of the study**	**Setting**	**Study population**	**Methods**	**Determinants and outcomes**
1	Juguan et al. (21)	204	To investigate nutrient intake, anthropometric and biochemical indicators, and their associations	Community two sub-villages of Johor-Baru Sub-district in Jakarta, Indonesia.	Non-institutionalized older adults between 60 and 75 years	Questionnaires FFQ Weight, height, arm span, skinfolds of biceps, triceps, supra-iliac, and sub-scapula BMI Hemoglobin, thiamine, retinol with liquid chromatography, vitamin B12	Socioeconomic, lifestyle, and health status Habitual food intake Anthropometric characteristics Biochemical characteristics
2	Purba et al. (22)	461	To identify differences in eating patterns and in food and energy intakes between older adults residing in urban metropolitan Jakarta and urban non-metropolitan Semarang to investigate the prevalence of food and energy deficiencies.	Community data from the Nutrition and metabolic Study of Indonesian Older adults (NUMSIE), 10 public health centers in both Jakarta and Semarang	Men and women aged 55 years and over from the public health centers. Only individuals aged 60 years and over were included in the analysis	Questionnaires: general health status, physical activity blood sample physical examination FFQ	Demography and health status Food habits
3	Kamso et al. (20)	556	To investigate determinants of blood pressure in older adults who differed in body composition	Community older adults clubs of 15 public health Centers in sub-districts of Jakarta, Indonesia	Individuals aged 55–80 years	24-h dietary recall Weight height, abdominal and hip circumferences, skinfold thickness of biceps, triceps, subscapular and supra-iliac Blood sample for cholesterol, triglycerides, fasting blood glucose, and sphygmomanometer for blood pressure	Nutritional status Anthropometric measures Blood measures
4	Arjuna et al. (23)	527	To determine the socio-demographic and anthropometric characteristics and the nutritional, health, mental and functional status of community-dwelling older men and women in Yogyakarta	Community rural (2 out of 12 sub-districts) and urban (2 out of 14 sub-districts) suburbs of Yogyakarta	Older (≥65 years) individuals living in Yogyakarta for the last year	Body weight, height, fat percentage, waist, hip, mid-arm, and calf circumference and triceps, biceps, sub-scapula, and supra-iliac skinfold thickness	Anthropometric characteristics
						MNA, SNAQ, GNRI, MMSE, GDS, grip strength, gait speed, IADL, IPAQ, FRAIL score, Blood analysis (blood count, albumin, CRP, cytokines	Nutritional, mental, and functional status, frailty status
						24-h food recall	Energy and nutrient intake
						SQ-FFQ	Food choices and sources of nutrients
5	Pengpid and Peltzer (24)	29.509 (3.585 are >60 year old)	To quantify the prevalence of underweight and overweight or obesity and its related factors	Community data from the Indonesian family life survey wave 5	Adults (>18 years) in Indonesia from enumeration areas. Some sub-group analyses are based on age, including >60 years old	Age, sex, marital status, education, work, religion, residential status, socioeconomic background	Socio-demographic characteristics
						Height, weight, BMI, FFQ	Nutritional status, food consumptions
						Questionnaire: childhood hunger, self-reported health status, physical activity, diabetes, hypertension, high cholesterol, sleep disturbances, happiness	Health status
						The “Patient-Reported Outcomes Measurement Information system (PROMIS)” sleep disturbance and sleep impairment measures	Sleep disturbance and sleep impairment
6	Mutiara et al. (25)	58	To evaluate the correlation between hair zinc level and cognitive function in older adults	Community kartini regency, central jakarta	Individuals aged 60 years and older	Interview: age, sex, education, work, medical history	Baseline characteristics
						Height, weight, calf circumference	Anthropometric measurements
7	Setiati et al. (26)	702	To obtain the cut-off value of anthropometric measurements and nutritional status of older adults in Indonesia (multi-center study)	Outpatient outpatient clinics of 10 hospitals in 10 cities in Indonesia	Individuals of 60 years and older attending the outpatient clinic of hospitals having no disability and chronic diseases	BMI, FFQ, MNA, hair sample zinc MNA, BMI, serum albumin	Nutritional status Nutritional status
8	Setiati et al. (8)	387	To obtain profile of food and nutrient intake and factors associated with energy intake	Outpatient geriatric outpatients of geriatric clinics in 15 cities in Indonesia	Older adults (aged 60 years and older)	Age sex, education, present activities, co-morbidities, BI, GDS, MMSE MNA 24-h food recall	Functional status and demographic data Nutritional status nutrient intake
9	Riviati et al. (27)	325	To determine the relationship between age, nutritional status, and chronic diseases with handgrip strength	Outpatient geriatric outpatient clinic of Cipto Mangunkusumo Hospital and Mohammad Hoesin Hospital	Patients aged above 60 years old with comorbidity or chronic disease, without mental problems or acute diseases	Age, sex, waist circumference, handgrip strength Stroke, hypertension, diabetes, coronary heart disease, COPD MNA	Anthropometric characteristics Chronic diseases Nutritional status

*BI, Barthel Index; MNA, Mini Nutritional Assessment; FTU, Functional Tooth Units; BMI, Body Mass Index; CRP, C-Reactive Protein; ELISA, Enzyme-Linked Immuno-Sorbent Assay; FFQ, Food Frequency Questionnaire; IL, Interleukin; IFN, Interferon; PTH, Parathyroid Hormone; MMSE, Mini-Mental State Examination; GDS, Geriatric Depression Scale; COPD, Chronic Obstructive Pulmonary Disease*.

### Risk of Bias Within Studies

The risk of bias was assessed using the Joanna Brigg's Institute (JBI) critical appraisal checklist for studies reporting prevalence data, attached as an Appendix. For every appraisal point, the methodological quality of the articles was assessed and summarized in Table 2. This table describes every appraisal point of validity, importance, and applicability of the target population of each study. The lowest point on the JBI critical appraisal form of ten checklist points in the JBI critical appraisal was five out of ten Kamso et al. and the highest point was ten Arjuna et al.

**Table 2:** Risk of bias assessed by the Joanna Brigg's Institute (JBI) critical appraisal checklist for studies reporting prevalence of malnutrition data.

**References setting**	**Representation of the target population**	**Recruitment appropriate**	**Sample size adequate**	**Subjects and setting described**	**Analysis sufficient coverage sample**	**Standard criteria for the measurement**	**Reliable measurement**	**Statistical analysis reliable**	**Confounding factors accounted**	**Subpopulation identified objectively**
Juguan et al. (21) community	Yes	Yes	Yes	Yes	Yes	Yes	Yes	Yes	Not applicable	Not applicable
Purba et al. (22) community	Yes	Yes	Yes	Yes	Yes	Yes	Yes	Yes	Not applicable	Not applicable
Kamso et al. (20) community	Yes	Unclear	Unclear	Yes	Yes	Yes	Yes	Unclear	Not applicable	Not applicable
Arjuna et al. (23) community	Yes	Yes	Yes	Yes	Yes	Yes	Yes	Yes	Yes	Yes
Pengpid and Peltzer (24) community	Yes	Yes	Yes	Yes	Yes	Unclear	Yes	Yes	Yes	Yes
Mutiara et al. (25) community	Yes	Yes	Yes	Yes	Yes	Unclear	Unclear	Yes	Not applicable	Not applicable
Setiati et al. (26) outpatient	Yes	Unclear	Unclear	Yes	Yes	Yes	Yes	Yes	Yes	Not applicable
Setiati et al. (8) outpatient	Yes	Yes	Yes	Yes	Yes	Yes	Yes	Unclear	Yes	No
Riviati et al. (27) outpatient	Yes	Yes	Unclear	Yes	Yes	Unclear	Unclear	Yes	Unclear	Yes

“*Yes” good methodological quality; “No” bad methodological quality; “Unclear” not clearly defined/further information required, “Not applicable” not relevant based on study design*.

### Results of Individual Studies

Most studies presented the prevalence of malnutrition based on BMI categories and MNA criteria. The prevalence of malnutrition is shown in Table 3 according to BMI categories and in Table 4 according to MNA categories. All studies in Table 3 used a BMI of 18.5 kg/m^2^ as a criterion for underweight, whereby underweight prevalence ranged from 8.0 to 26.6%. According to the MNA, the prevalence of malnutrition ranged from 2.1 to 14.6%, whereby the prevalence of at risk of malnutrition amounted from 18 to 78%.

**Table 3:** Prevalence of malnutrition based on BMI categories in older adults.

**References**	**N**	**Setting**	**BMI classification (kg/m^2^)**	**Prevalence (%)**
Juguan et al. (21)	204	Community	Underweight (<18.5)	26.6%
			Overweight (>25.0)	12.3%
Kamso et al. (20) (Based on WHO criteria)	556	Community	Underweight	8.0%
			Normal weight	50.0%
			Overweight	30.0%
			Obese	>5%
Pengpid and Peltzer (24)	3.585	Community	Underweight (<18.5)	20.9%
			Normoweight	42.2%
			Overweight/obesity	36.9%
Mutiara et al. (25)	58	Community	Underweight (<18.5)	15.5%
			Normal range (18.5–22.9)	39.7%
			Overweight (23.0–24.9)	13.8%
			Obese I (25.0–29.9)	25.9%
			Obese II (>30.0)	5.1%
Setiati et al. (26)	702	Outpatient	Underweight (<18.5)	10.4%
			Normal weight (18.5–22.9)	45.0%
			Overweight (23–24.9)	22.5%
			Obese (≥25.0)	22.1%

**Table 4:** Prevalence (percentage of total population) of malnutrition in older adults based on MNA (Mini Nutritional Assessment) criteria.

**References**	**Sub population (if applicable)**	***N***	**Setting**	**MNA criteria**	**Prevalence (%)**
Arjuna et al. (23)	Rural	302	Community	Malnourished	3.0%
		324		At risk of malnutrition	73.0%
	Urban	302		Malnourished	6.0%
		324		At risk of malnutrition	44.0%
Mutiara et al. (25)*		13	Community	at risk of malnutrition	77.6%
		45		Normal nutritional status	22.4%
Setiati et al. (26)		702	Outpatient	Malnourished	2.1%
				At risk of malnutrition	56.7%
				Well-nourished	41.2%
Setiati et al. (8)		387	Outpatient	Malnourished	5.2%
				At risk of malnutrition	17.6%
				Normal nourished	69.3%
Riviati et al. (27)		325	Outpatient	Malnourished	14.6%
				Normal nutritional status	86.4%

* *MNA short form*.

Data on the level and distribution of energy and nutrient intakes (Table 5) were extracted from six papers, five published papers, and one gray literature paper (25). Unfortunately, none of the studies reported validation of the questionnaires or the full spectrum of nutrients. Only data on nutrients that are available presented in Table 5. Juguan (21) reported data on energy, protein, and vitamin B12 intake only. Purba (22) reported data on food and food groups using an FFQ, yet they included only data on energy intake. Pengpid (24), the study with the largest sample size, used FFQ as part of measurements, but they only presented food intake and did not convert it into nutrient intake. Kamso (20), Setiati (8), and Arjuna (23) report data on almost all nutrients. Mutiara et al. (25) analyzed their raw data and reported data on energy and all nutrients intake as gray literature. We converted the values of the standard error of nutrient intake in studies of Kamso (20) and Setiati (8) into the values of standard deviation to make the data in Table 5 more comparable and available for further analysis.

**Table 5:** Data on the level and distribution of habitual intake of energy, macronutrients and a selection of micronutrients.

**References**	**Dietary assessment method**	**Setting**	**Sub population**	***N***	**Energy (kcal)**	**Protein (g)**	**Carbohydrate (g)**	**Fat (g)**	**SFA (g)**	**MUFA (g)**	**PUFA (g)**	**Calcium (mg)**	**Vitamin D (μg)**	**Vitamin B12 (μg)**
Juguan et al. (21) Median (P25–75)	FFQ	Community	Men	93	1,416 (1,120–1,739)	43.2 (34.2–50.3)	Not reported	22.1 (15.5–32.0)	Not reported	Not reported	Not reported	Not reported	Not reported	3.5 (2.0–5.9)
			Women	111	1,251 (989–1,521)	37.6 (30.1–48.1)	Not reported	20.9 (14.1–33.0)	Not reported	Not reported	Not reported	Not reported	Not reported	3.0 (1.7–5.7)
Purba et al. (22) range	FFQ	Community	Jakarta older adults	212	1,251–2,079	Not reported	Not reported	Not reported	Not reported	Not reported	Not reported	Not reported	Not reported	Not reported
			Semarang older adults	238	939–1,579	Not reported	Not reported	Not reported	Not reported	Not reported	Not reported	Not reported	Not reported	Not reported
Kamso et al. (20) mean ± SD (calculated from SE)	24-h food recall	Community	Men	161	1,257 ± 419	51.6 ± 43.2	170 ± 52.1	42.9 ± 21.6	Not reported	Not reported	Not reported	397 ± 269.2	Not reported	Not reported
			Women	395	1,090 ± 398	43.1 ± 23.9	143 ± 51.7	39.2 ± 21.9	Not reported	Not reported	Not reported	379 ± 260.7	Not reported	Not reported
Arjuna et al. (23) mean ± SD	24-h recall, FFQ	Community	Urban men	132	1,530 ± 500	45 ± 21	229 ± 72	51 ± 24	31 ± 22	9 ± 6	6 ± 4	352 ± 244	1.1 ± 2.8	Not reported
			Urban women	192	1,365 ± 445	40 ± 18	197 ± 64	48 ± 22	29.6 ± 29.3	8 ± 5	6 ± 4	346 ± 207	1.0 ± 2.6	Not reported
			Rural men	83	1,520 ± 447	39 ± 15	237 ± 75	48 ± 21	28.3 ± 12.9	7 ± 4	6 ± 5	309 ± 135	1.0 ± 2.3	Not reported
			Rural women	120	1,278 ± 402	34 ± 13	186 ± 60	46 ± 22	26 ± 12	8 ± 7	6 ± 6	301 ± 181	0.9 ± 2.0	Not reported
Mutiara et al. (25) Mean ± SD	FFQ	Community		58	1,686 ± 509	63.1 ± 26.1	260.6 ± 87.9	45.0 ± 19.6	Not reported	Not reported	Not reported	489 ± 295	4.1 ± 5.1	4.3 ± 6.4
Setiati etal. (8) mean ± SD (calculated from SE)	24-h food recall	Outpatient	All participants	387	1,267 ± 337	44.7 ± 15.5	172.0 ± 57.9	41.2 ± 17.9	Not reported	Not reported	Not reported	Not reported	Not reported	Not reported
			Men	161	1,313 ± 341	45.3 ± 13.4	184 ± 59.6	41 ± 25.4	Not reported	Not reported	Not reported	257 ± 27.9	Not reported	Not reported
			Women	226	1,234 ± 330	44.3 ± 22.5	164 ± 72.2	42 ± 25.5	Not reported	Not reported	Not reported	267 ± 33.1	Not reported	Not reported

Data to derive the prevalence of inadequacies of energy and nutrients intake could be extracted from five papers, four published papers, and one gray literature paper (25). We calculated the prevalence of intakes below the RDA and 2/3 RDA as the value of estimated EAR based on available data of studies in Table 5 and present those by gender are shown in Table 6. Purba (22) presented the energy intake as a range, so we could not calculate the prevalence of inadequacies. Juguan (21) presented nutrient intake data as medians with the corresponding 25 and 75 percentiles. Therefore, we could not estimate the prevalence of inadequacies either. Mutiara et al. (25) reported nutrient intake in the total population regardless the gender. Consequently, we were able to calculate the prevalence of inadequacies for calcium, vitamin D, and vitamin B12 only. Compared with the Indonesian RDA, intakes of most nutrients are below recommendation for most individuals. Using 2/3^*^RDA as a proxy of the EAR, estimated proportions of inadequacies were highest for vitamin D and Calcium, followed by protein and vitamin B12 (Table 6). When comparing the intakes with 2/3 RDA, the prevalence of inadequacies remained over 40% for protein and vitamin B12 intakes and over 90% for vitamin D and calcium among Indonesian older adults.

**Table 6:** Prevalence of energy, macronutrients, and a selection of Micronutrients Intakes below RDA and Estimated EAR.

**References**	**Setting**	**Sub group**	**Energy**		**Carbohydrates**		**Fat**		**Protein**		**Calcium**		**Vitamin D**		**Vitamin B12**	
**RDA values**			***RDA***	***2/3 RDA***	***RDA***	***2/3 RDA***	***RDA***	***2/3 RDA***	***RDA***	***2/3 RDA***	***RDA***	***2/3 RDA***	***RDA***	***2/3 RDA***	***RDA***	***2/3 RDA***
		Men	1,800 kcal	1,200 kcal	275 g	183 g	50 g	33 g	64 g	43 g	1,200 mg	800 mg	20 μg	13 μg	4 μg	3 μg
		Women	1,550 kcal	1,034 kcal	230 g	153 g	45 g	30 g	58 g	39 g						
			*% below RDA*	*% below 2/3 RDA*	*% below RDA*	*% below 2/3 RDA*	*% below RDA*	*% below 2/3 RDA*	*% below RDA*	*% below 2/3 RDA*	*% below RDA*	*% below 2/3 RDA*	*% below RDA*	*% below 2/3 RDA*	*% below RDA*	*% below 2/3 RDA*
Kamso et al. (20)	Community	Men	90.1	61.0	97.8	59.9	62.9	32.3	61.4	42.0	99.9	93.1	Not available	Not available	Not available	Not available
		Women	87.7	44.4	95.4	57.5	60.3	33.7	73.2	43.3	99.9	94.6	Not available	Not available	Not available	Not available
Arjuna et al. (23)	Community	Men (urban)	70.5	25.5	73.6	26.1	49.0	22.7	70.0	46.4	99.9	96.7	>99.9	>99.9	Not available	Not available
		Men (rural)	73.6	23.6	69.5	46.4	53.6	23.9	95.3	60.6	>99.9	99.9	>99.9	>99.9	Not available	Not available
		Women (urban)	66.3	23.0	69.1	24.5	13.6	20.6	84.0	48.0	>99.9	98.6	>99.9	>99.9	Not available	Not available
		Women (rural)	70.5	27.1	76.7	29.1	48.0	23.3	96.8	64.8	>99.9	99.7	>99.9	>99.9	Not available	Not available
Mutiara et al. (25)	Community		Not available	Not available	Not available	Not available	Not available	Not available	Not available	Not available	99.2	85.3	99.9	95.9	48.0	42.0
Setiati et al. (8)	Outpatient	Men	92.2	37.1	99.9	48.4	63.3	37.8	91.9	43.3	>99.9	>99.9	Not available	Not available	Not available	Not available
		Women	82.6	27.1	99.5	33.4	54.8	31.9	72.9	40.3	>99.9	>99.9	Not available	Not available	Not available	Not available

*Moderate prevalence of inadequate nutrient intake (>33–66% below estimated EAR) presented in blue cells, while severe inadequacy of nutrient intake (>66% below estimated EAR) presented in red cells*.

## Discussion

Our systematic review identified a high prevalence of malnutrition risk in Indonesian community-dwelling older adults, with suboptimal energy and nutrients intakes, most markedly for protein, calcium, vitamin D, and vitamin B12. To our knowledge, this study is the first systematic review reporting on the prevalence of malnutrition and inadequate intake of nutrients in Indonesian older adults. Applying a systematic approach and evaluating study quality and risk of bias further strengthens the results. For the time being, a systematic review is the best available option now that large surveys are not foreseen. A systematic review like ours is of particular relevance where individual studies are limited by small sample sizes. It uniquely provides a clear and comprehensive overview of the available evidence on the prevalence of both malnutrition and nutritional inadequacies in Indonesian older adults.

The target population of this systematic review concerned community-dwelling elderly people in Indonesia. Although we found limited data to answer our review questions, we could review data collected from nine studies. Assessing the nine included studies in this review by the JBI criteria, the study populations in all included articles appeared good samples of community-dwelling older adults as the target population. Therefore, there is good reason to assume that our results can be extrapolated to the general, apparently healthy Indonesian older adults. The choice to include only Indonesian elderly populations was made to have national data on malnutrition and nutrient inadequacies. It may have affected the external validity of our findings. Yet, our results might be extrapolated to other lower-middle-income countries (LMICs) with comparable characteristics and dietary patterns.

The inception of this systematic review started before the concept of the Global Leadership Initiative on Malnutrition (GLIM) criteria for diagnosis of malnutrition was introduced in 2019 (28). Therefore, studies included in this systematic review did not apply the updated GLIM criteria for malnutrition, and the definitions of malnutrition relied on BMI and MNA criteria. Differences in methods to assess dietary intake assessment, including food recall and FFQ, must be considered in interpreting the results. The methods might underestimate the actual intakes due to impairment of memory and other functional skills (vision, hearing, and writing ability) among older adults. According to Vries et al., dietary assessment applied in community-dwelling elderly people underestimated their intake up to 10–15% compared with the reference value (29). Thus, the prevalence of inadequate intake of energy and nutrient might be overestimated.

We found prevalence of malnutrition ranging from 8 to 26% (according to BMI), or 2–15% (according to MNA). Hereby, we acknowledge that in most of the studies reviewed, the prevalence of malnutrition is either below 16% (six out of seven studies using the BMI criterion) or below 5% (four out of five studies using MNA criterion). Yet, in most studies, the risk of malnutrition was pronounced, amounting to over 44% in four out of five studies, including the MNA. Prevalences found in the literature are well within these ranges or even higher. In Singapore, the prevalence of malnutrition using the Mini Nutritional Assessment (MNA) in community-dwelling people aged **≥**65 years was 3.6% (30). Another community cross-sectional study done in Uttarakhand, India, by Gupta et al. found that the prevalence of underweight was 26.6% (31). Wei et al. in China showed that malnutrition in older adults (age of >60 years old) was 12.6% based on BMI (32). Two studies conducted in India found that the prevalence of malnutrition based on MNA was higher than in this review. The studies conducted by Mathew et al. found that the malnutrition prevalence amounted to 19.5% (33) while the study done by Krishnamoorthy et al. found a prevalence of 17.9% (34). Among Asian countries, Vietnam has the highest prevalence of malnutrition (42.7%) among older adults with multimorbidity (35). This comparison points toward the high risk of malnutrition in community-dwelling populations in most Asian countries.

We observed a wide variability in the intake of energy and nutrients and a high prevalence of inadequacies for all dietary intake parameters compared with RDA values and with specific nutrients compared to the EAR estimates. Nutrient standards that specify a mean requirement, such as the EAR, are preferably used to estimate the prevalence of nutrient adequacy for a group (15, 16). This approach to assessing dietary nutrient intakes is considered more informative than former methods, which relied on the RDA, overestimating the prevalence of inadequacies (36). If sufficient scientific evidence is not available to set an EAR, a calculation using formula 2/3 RDA can be used as a proxy for the EAR (17). According to a study conducted by Ong et al., information on dietary and nutrient intakes is relatively scarce in Asia. Most of the data, particularly for older adults, were obtained through small cohort studies and limited due to the community level setting. Furthermore, RDAs rather than EARs were used as reference values (14). Most Asian countries derived their recommended daily intakes or allowances (RDI or RDA) from national nutrition surveys, small scale surveys, and/or household food consumption findings, following the framework established by the Institute of Medicine's Food and Nutrition Board, which aimed to meet the requirements of 97.5 percent of healthy individuals by life stage and gender. The reported mean intake values were generally compared to the RNI/RDA. The reported values did not include proportions that were lower than the estimated average recommendation (EAR), which is now used globally. Several countries are currently updating their reference values. The vast majority of countries agree that these recommendations should be updated (14).

We found limited data to compare our data with studies in Southeast Asian countries, so we compared our findings to those of other Asian countries. A literature review conducted by Park et al. in Korean older adults found that the average total intake of energy was between 7,700 and 8,800 KJ/day (1,842–2,105 kcal/day). They reported inadequate intake of several micronutrients, mostly calcium and vitamin A (37). Therefore, we compared our findings with systematic reviews in the western population. The high prevalence of energy and macronutrients inadequacies are in line with the study conducted by ter Borg et al., a systematic review of macronutrient intake and inadequacies of community-dwelling older adults in western populations (Europe, North America, Australia, New Zealand) (11). The difference of our study with ter Borg et al. study is only in fat intake and that of specific micronutrients. The fat intake was at the upper end of the reference value in the western population. Regarding micronutrient inadequacies, almost all older adults in the Indonesian community-dwelling population have inadequate calcium and vitamin D intakes, even when we compared the intakes to 2/3 RDA. The prevalence of inadequate vitamin B12 intake is 48 and 42%, based on RDA and 2/3 RDA, respectively. The result of our study for the prevalence of vitamin D inadequacy is more than 95%. Vitamin D deficiency has been identified as a worldwide problem (37). Ter Borg also reported inadequacies in particular for vitamin D in western older adults. Yet, our population's prevalence of calcium inadequacies is much higher (more than 95%) compared with the western population (65% for men and 73% for women). The difference of findings also concerns the prevalence of vitamin B12 inadequacy, revealed 42% in the Indonesian population. In contrast, in the western population, the prevalence is much lower (16% for men and 19% for women) (38). This “east meets west” comparison indicates that inadequacies of energy and macronutrients intakes might be a global problem, but the inadequacies of micronutrients intakes are worse in Asian countries, e.g., Indonesia.

Whether these low nutrient intakes are of true public health concern and the other nutrients are not depends on several factors. In older adults, the picture of nutritional status is not complete without also considering clinical assessment, nutrient absorption, utilization as assessed by biochemical status, micronutrient supplementation use, and potential differences in the nutrient requirements/recommendations upon which the percentage at risk calculation is based (38). Reduction of energy intake may also result in decreased intake of other nutrients, resulting in nutrient deficiencies. Nutrient requirements, however, do not necessarily decrease with age and even tend to increase. The need for protein might increase because of age-related conditions such as loss of muscle mass and anabolic resistance (39). For protein intake, the reference values for older adults are a topic of continuous debate. A higher recommended level for older adults is argued by protein experts in the field (39). The high prevalence of vitamin D deficiency has been a worldwide concern. A high proportion of the population has low intakes of vitamin D because dietary sources are rare and limited to oily fish such as salmon, mackerel, and sardines are rich in vitamin D3. Egg yolks are reported to contain vitamin D though the amounts are highly variable, and in some cases, dairy products. Most of the vitamin D we use is delivered through skin synthesis and/or dietary supplements (40). Although Indonesia is a tropical country, with high sun exposure all year around, there might be genetic *BsmI* polymorphisms in the vitamin D receptor gene in the Indonesian–Malay race, contributing to the high prevalence of vitamin D inadequacy (41).

Vitamin D deficiencies have been related to fractures, falls, and low physical performance and potentially also to age-related cognitive decline. Higher dietary and supplementary intakes of vitamin D result in the reversal of vitamin D deficiencies and increased serum 25 (OH) D concentrations among community-dwelling older adults. The functional outcome of Ca intake is often bone density, where higher intakes of Ca (500 mg/d) plus vitamin D3 are associated with a higher bone density. However, calcium absorption is dependent on vitamin D intake because vitamin D facilitates the intestinal absorption of calcium. While biomarkers for calcium are generally thought to be problematic because they have no specific proper measurement technique, the blood level of vitamin d 25 (OH) D has been routinely checked in clinical practice (40). Concerning the inadequacy of vitamin B12 intake in the Indonesian elderly population, this problem might be due to the dietary pattern. The usual dietary sources of Vitamin B12 are animal-derived foods such as meat, milk, egg, fish, and shellfish. Thus, it is necessary to identify plant-derived food sources that naturally contain a large amount of Vitamin B12 to prevent Vitamin B12 deficiency in a population at risk (42). The bioavailability of vitamin B12 in healthy humans from fish meat, sheep meat, and chicken meat averaged 42, 56–89%, and 61–66%, respectively. Vitamin B12 in eggs seems to be poorly absorbed (<9%) relative to other animal food products (43). Another issue related to vitamin B12 deficiency is malabsorption of vitamin B12 among older adults because atrophy of the gastric folds impairs gastric acid production. Even high intakes of vitamin B12 from dietary and supplementary sources have a plateau effect in increasing serum concentrations because there is less efficient absorption with higher intakes. In the general population, some studies have shown that among patients with anemia, ~1–2% is due to B12 deficiency. The anemia then leads to symptoms such as fatigue and pallor that are commonly seen in patients with B12 deficiency. Low levels of vitamin B12 also have been linked with an increased risk of fractures, and less robust evidence exists for a relationship between vitamin B12 status and cognitive function (44), as well as homocysteine level-associated bone fracture (45). Therefore, the present systematic review results have to take into consideration the interplay of several factors.

Inadequacies of nutrient intake are determinants of malnutrition. Malnutrition leads to various health problems, mainly frailty, morbidity, and mortality. Nutrition interventions might effectively treat these conditions (46). Development of country-specific food-based dietary guidelines and updating the national dietary reference intakes are recommended. Further insights into the nutrient intake and diet quality of older adults may contribute to healthy aging and improve their quality of life (47). In clinical practice, one of the recommendations is to encourage all general practitioners to screen for malnutrition risk as well as dietary intake from the first time they see an elderly patient, thereby preventing malnutrition even further (7). Nutrition should be incorporated into any program or initiative aimed at improving health outcomes. A healthy diet may result in a lower mortality rate. If the recommended intake of 20 g (800 IU) of vitamin D per day is met, it can lead to a 20–28% reduction in fractures and falls. A higher protein intake (>25 g/meal) combined with twice weekly resistance exercise increases lean muscle mass. As a result, a healthy dietary pattern and nutrition education should be incorporated into programs and initiatives to ensure adequate nutrient intake combined with optimal lifestyle habits such as physical activity. These elements are critical for disease prevention and health outcomes improvement (12). Policy makers in low- and middle-income countries with a growing elderly population may be able to use our study's findings to encourage development in healthcare for the elderly (7). Furthermore, a large population-based survey would beneficial. In fact, national data on the nutritional status of Indonesian older adults are being collected. The Indonesian Geriatrics Society (PERGEMI) actively seeks support for the survey from the Indonesian Ministry of Health. In such future studies, we would acknowledge relevant advances in malnutrition consensus statements as proposed by the Global Leadership Initiative on Malnutrition (GLIM) in 2019.

In conclusion, we signal a high risk of malnutrition in Indonesian older adults along with poor macronutrients and micronutrients intakes. The present review provides important and robust evidence of the magnitude of the malnutrition problem and nutrient intake concerns among Indonesian community-dwelling older adults. The promotion of nutrient-rich foods consumption needs to be explored. Strategies to tackle malnutrition and inadequacies of energy and nutrients intakes are most relevant to open the door for more nutrient-dense foods in the diet of Indonesian older adults.

## Data Availability Statement

The original contributions presented in the study are included in the article/Supplementary Material, further inquiries can be directed to the corresponding author/s.

## Author Contributions

ED, RA, SFS, AP, LG, and SS contributed to development of study concept and design. SFS and AP contributed to acquisition of data. ED, LG, FH, MK, and SS contributed to analysis and interpretation of data. ED, RA, SFS, AP, FH, MK, LG, and SS contributed to drafting of the manuscript. All authors contributed to the article and approved the submitted version.

## Funding

The funding source of this study was supported by a grant from the Judith Zwartz Foundation, Wageningen, the Netherlands (VLAG Scholarship, Project No. 6130011310).

## Conflict of Interest

The authors declare that the research was conducted in the absence of any commercial or financial relationships that could be construed as a potential conflict of interest.

## Publisher's Note

All claims expressed in this article are solely those of the authors and do not necessarily represent those of their affiliated organizations, or those of the publisher, the editors and the reviewers. Any product that may be evaluated in this article, or claim that may be made by its manufacturer, is not guaranteed or endorsed by the publisher.
