# Supplementary material for: Malnutrition Prevalence and Nutrient Intakes of Indonesian Community-Dwelling Older Adults: A Systematic Review of Observational Studies
Source: Front Nutr. 2022 Feb 24;9:780003. doi: 10.3389/fnut.2022.780003 (PMC8912970; doi:10.3389/fnut.2022.780003)
Supplement: Supplementary file 1 [file Data_Sheet_1.docx]

Supplementary Material

**Appendix 1.** Definitions of data items based on database

| **Database** | **Search strategy** |
| --- | --- |
| MEDLINE | (((((((((((((((older[Title/Abstract]) OR elderly[Title/Abstract]) OR elder[Title/Abstract]) OR frail[Title/Abstract]) OR frailty[Title/Abstract]) OR geriatric[Title/Abstract]) OR "older person"[Title/Abstract]) OR "older adults"[Title/Abstract]) OR frail elderly[MeSH Terms]) OR (aged, 80 and over[MeSH Terms])) OR geriatrics[MeSH Terms]))) AND ((((((((((((((((((malnutrition[MeSH Terms]) OR malnutrition, protein energy[MeSH Terms]) OR malnutrition, protein calorie[MeSH Terms]) OR cachexia[MeSH Terms]) OR undernutrition[MeSH Terms]) OR underweight[MeSH Terms]) OR malnutrition[Title/Abstract]) OR "protein energy"[Title/Abstract]) OR cachexia[Title/Abstract]) OR undernutrition[Title/Abstract]) OR underweight[Title/Abstract]) OR malnourished[Title/Abstract]) OR "low BMI"[Title/Abstract]) OR anorexia[MeSH Terms]) OR anorexia[Title/Abstract]) OR under-nutrition[Title/Abstract]) OR cachexic[Title/Abstract])))) AND Indonesia |
| CENTRAL | MeSH descriptor: [Aged, 80 and over] explode all trees  OR  MeSH descriptor: [Frail Elderly] explode all trees  OR  MeSH descriptor: [Frailty] explode all trees  OR  ((geriatric OR older OR elderly OR elder OR frail OR frailty OR "older person" OR "older adults")): ti,ab,kw  AND  MeSH descriptor: [Malnutrition] explode all trees  OR  MeSH descriptor: [Protein-Energy Malnutrition] explode all trees  OR  MeSH descriptor: [Cachexia] explode all trees  OR  MeSH descriptor: [Thinness] explode all trees  OR  MeSH descriptor: [Anorexia] explode all trees  OR  ((malnutrition OR cachexia OR "protein energy" OR undernutrition OR underweight OR malnourished OR BMI OR anorexia OR cachexic OR under-nutrition)): ti,ab,kw  AND  Indonesia |
| EMBASE | (older or elderly or elder or frail or frailty or geriatric or "older person" or "older adults"). ab.  AND  (malnutrition or cachexia or "protein energy" or undernutrition or underweight or malnourished or BMI or anorexia or cachexic or under-nutrition). ab.  AND  Indonesia |
| ProQuest | ab(geriatric OR older OR old OR aging OR aged OR elderly OR elder OR frail OR frailty OR frailties OR senior OR "older person" OR "older adults") AND ab(Indonesia) AND (malnutrition OR "protein energy" OR cachexia OR undernutrition OR underweight OR malnourished OR "low BMI" OR anorexia OR under-nutrition OR cachexic) |

**Appendix 2.** Joanna Briggs Institute Appraisal Checklist for Studies Reporting Prevalence Data


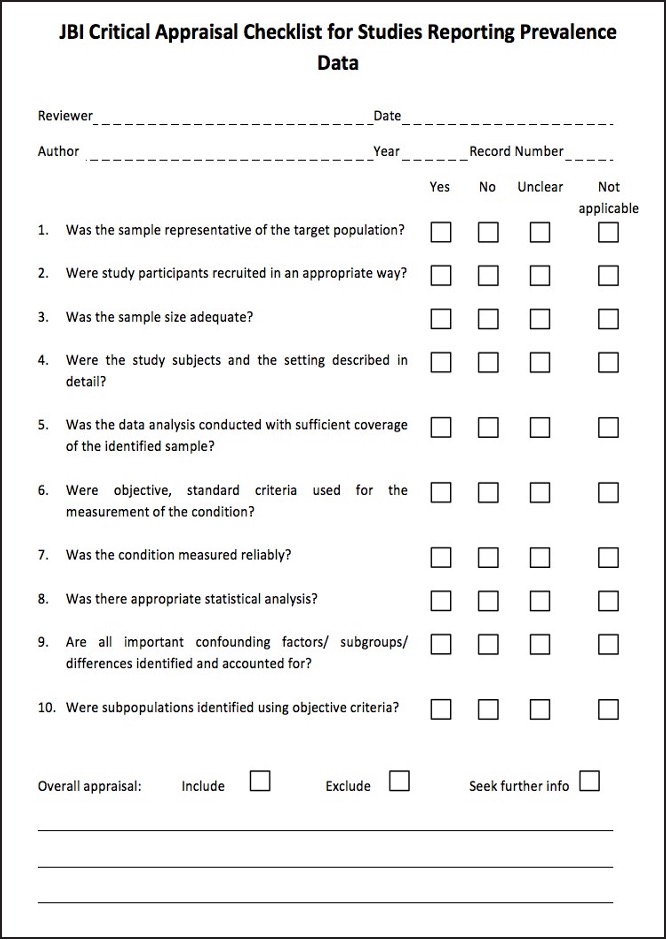


**Prevalence Critical Appraisal Tool**

Answers: Yes, No, Unclear or Not/Applicable

1. Was the sample representative of the target population?

This question relies upon knowledge of the broader characteristics of the population of interest. If the study is of women with breast cancer, knowledge of at least the characteristics, demographics and medical history is needed. The term “target population” should not be taken to infer every individual from everywhere or with similar disease or exposure characteristics. Instead, give consideration to specific population characteristics in the study, including age range, gender, morbidities, medications, and other potentially influential factors. For example, a sample may not be representative of the target population if a certain group has been used (such as those working for one organization, or one profession) and the results then inferred to the target population (i.e., working adults).

1. Were study participants recruited in an appropriate way?

Recruitment is the calling or advertising strategy for gaining interest in the study, and is not the same as sampling. Studies may report random sampling from a population, and the methods section should report how sampling was performed. What source of data were study participants recruited from? Was the sampling frame appropriate? For example, census data is a good example of appropriate recruitment as a good census will identify everybody. Was everybody included who should have been included? Were any groups of persons excluded? Was the whole population of interest surveyed? If not, was random sampling from a defined subset of the population employed? Was stratified random sampling with eligibility criteria used to ensure the sample was representative of the population that the researchers were generalizing to?

1. Was the sample size adequate?

An adequate sample size is important to ensure good precision of the final estimate. Ideally, we are looking for evidence that the authors conducted a sample size calculation to determine an adequate sample size. This will estimate how many subjects are needed to produce a reliable estimate of the measure(s) of interest. For conditions with a low prevalence, a larger sample size is needed. Also consider sample sizes for subgroup (or characteristics) analyses, and whether these are appropriate. Sometimes, the study will be large enough (as in large national surveys) whereby a sample size calculation is not required. In these cases, sample size can be considered adequate.

When there is no sample size calculation and it is not a large national survey, the reviewers may consider conducting their own sample size analysis using the following formula:

$$n=\frac{Z^{2}\times P(1-P)}{d^{2}}$$

Where:

n = sample size

Z = Z statistic for a level of confidence

P = Expected prevalence or proportion (in proportion of one; if 20%, P = 0.2)

d = precision (in proportion of one; if 5%, d=0.05)

1. Were the study subjects and setting described in detail?

Certain diseases or conditions vary in prevalence across different geographic regions and populations (e.g., women vs. men, sociodemographic variables between countries). Has the study sample been described in sufficient detail so that other researchers can determine if it is comparable to the population of interest to them?

1. Is the data analysis conducted with sufficient coverage of the identified sample?

A large number of dropouts, refusals or “not founds” amongst selected subjects may diminish a study’s validity, as can low response rates for survey studies.

- Did the authors describe the reasons for non-response and compare persons in the study to those not in the study, particularly with regards to their socio-demographic characteristics?
- Could the not-responders have led to an underestimate of prevalence of the disease or condition under investigation?
- If reasons for non-response appear to be unrelated to the outcome measured and the characteristics of non-responders are comparable to those in the study, the researchers may be able to justify a more modest response rate.
- Did the means of assessment or measurement negatively affect the response rate (measurement should be easily accessible, conveniently timed for participants, acceptable in length and suitable in content).

1. Were objective, standard criteria used for measurement of the condition?

Here we are looking for measurement or classification bias. Many health problems are not easily diagnosed or defined and some measures may not be capable of including or excluding appropriate levels or stages of the health problem. If the outcomes were assessed based on existing definitions or diagnostic criteria, then the answer to this question is likely to be yes. If the outcomes were assessed using observer reported, or self-reported scales, the risk of over- or under-reporting is increased, and objectivity is compromised. Importantly, determine if the measurement tools used were validated instruments as this has a significant impact on outcome assessment validity.

1. Was the condition measured reliably?

Considerable judgment is required to determine the presence of some health outcomes. Having established the objectivity of the outcome measurement instrument (see item 6 of this scale), it is important to establish how the measurement was conducted. Were those involved in collecting data trained or educated in the use of the instrument/s? If there was more than one data collector, were they similar in terms of level of education, clinical or research experience, or level of responsibility in the piece of research being appraised?

- Has the researcher justified the methods chosen?
- Has the researcher made the methods explicit? (For interview method, how were interviews conducted?)

1. Was there appropriate statistical analysis?

As with any consideration of statistical analysis, consideration should be given to whether there was a more appropriate alternate statistical method that could have been used. The methods section should be detailed enough for reviewers to identify the analytical technique used and how specific variables were measured. Additionally, it is also important to assess the appropriateness of the analytical strategy in terms of the assumptions associated with the approach as differing methods of analysis are based on differing assumptions about the data and how it will respond. Prevalence rates found in studies only provide estimates of the true prevalence of a problem in the larger population. Since some subgroups are very small, 95% confidence intervals are usually given.

1. Are all important confounding factors/ subgroups/differences identified and accounted for?

Incidence and prevalence studies often draw or report findings regarding the differences between groups. It is important that authors of these studies identify all important confounding factors, subgroups and differences and account for these.

1. Were subpopulations identified using objective criteria?

Objective criteria should also be used where possible to identify subgroups (refer to question 6).

**Appendix 3.** The Joanna Brigggs Institute’s data extraction form for prevalence studies


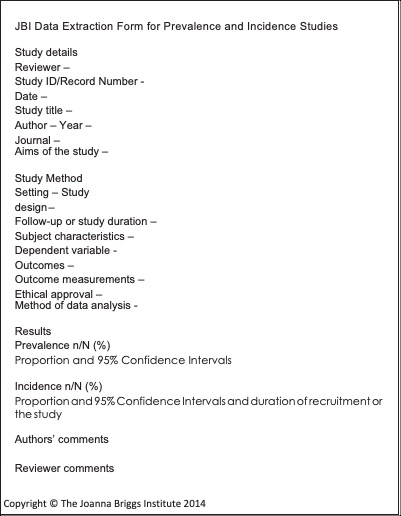


**Appendix 4.** The Indonesian Recommended Dietary Allowance (RDA) for age group 65-80 years old

| **Subpopulation** | **Reference** | **Energy (kcal)** | **Carbohydrate (g)** | **Protein (g)** | **Fat (g)** | **Calcium (mg)** | **Vitamin D (µg)** | **Vitamin B12 (µg)** |
| --- | --- | --- | --- | --- | --- | --- | --- | --- |
| Male | RDA | 1800 | 275 | 64 | 50 | 1200 | 20 | 4 |
|  | 2/3 RDA | 1200 | 183 | 43 | 33 | 800 | 13 | 3 |
| Female | RDA | 1550 | 230 | 58 | 45 | 1200 | 20 | 4 |
|  | 2/3 RDA | 1034 | 153 | 39 | 30 | 800 | 13 | 3 |
